# From Bioinformatics Analysis to Recombinant Expression: Advancing Public Health with *Taenia solium* Proteins

**DOI:** 10.3390/ijms26199585

**Published:** 2025-10-01

**Authors:** Juana Muñoz, María Camila Jurado Guacaneme, Clemencia Ovalle-Bracho, Julián Trujillo Trujillo, Sofía Duque-Beltrán, Adriana Arévalo, Carlos Franco-Muñoz

**Affiliations:** 1 Faculty of Sciences, Pontificia Universidad Javeriana, Bogotá, D.C. 110231, Colombia; camila.munoz2410@gmail.com (J.M.); mjurado@ins.gov.co (M.C.J.G.); clemencia-ovalleb@javeriana.edu.co (C.O.-B.); 2 Parasitology Group, Directorate of Public Health Research, Instituto Nacional de Salud, Bogotá, D.C. 111321, Colombia; sofiaduquebeltran@gmail.com (S.D.-B.); aarevalo@ins.gov.co (A.A.); 3 Emerging, Re-Emerging and Neglected Diseases Group, Sub-Directorate of Communicable Diseases, Ministry of Health and Social Protection, Bogotá, D.C. 110311, Colombia; jtrujillot@minsalud.gov.co

**Keywords:** *Taenia solium*, computational biology, recombinant proteins, immunodiagnosis

## Abstract

Taeniasis and neurocysticercosis (NCC), caused by *Taenia solium*, are significant public health concerns recognised by the World Health Organization (WHO) in developing countries across the Americas, Asia, and Africa. Taeniasis occurs in humans after consuming undercooked pork containing the larval stage (*Cysticerci*), which matures into the adult reproductive form in the intestine, releasing eggs through faeces. Accidental ingestion of these eggs by humans is the primary cause of NCC, a principal contributor to acquired epilepsy in endemic regions. Interrupting this transmission cycle is crucial to reducing the incidence of human NCC and porcine cysticercosis, thereby underscoring the need for accurate diagnosis and timely treatment of taeniasis. Current diagnostic tests for taeniasis, including microscopy, serology, copro-DNA, and coproantigen assays, exhibit variability in sensitivity, reproducibility, cross-reactivity, and accessibility. To overcome these limitations, bioinformatics tools were integrated with recombinant DNA technology to identify protein sequences with immunological potential. These sequences were evaluated in silico and used to construct an expression system. Subsequently, the antigens were expressed in a eukaryotic system, yielding two purified recombinant protein variants of 21 and 30 kDa. Their purification validated via Western blotting of the molecular tag, paves the way for the development of a direct immunological assay for the specific detection of *Taenia solium* carriers.

## 1. Introduction

Taeniasis is a foodborne zoonotic disease caused by the adult tapeworms *Taenia solium*, *Taenia saginata*, and *Taenia asiatica*. It has been classified as a neglected tropical disease by the World Health Organization (WHO) since 2010. *Taenia solium*, widely known as the “pork tapeworm”, is acquired through the consumption of undercooked pork containing cysts harbouring the larval stage (*cysticerci*). These larvae mature into the adult reproductive form within the human intestine, where they release eggs in the faeces. In the majority of cases, the infection remains asymptomatic. The parasite is endemic in developing countries where free-ranging pig farming and inadequate sanitation facilitate the completeness of its life cycle [1,2].

The cysticerci of *T. solium* are the causative agents of cysticercosis, which results from the ingestion of eggs released by infected individuals, either through direct faecal–oral contamination, contaminated food, or other indirect routes [3,4,5]. Neurocysticercosis (NCC), the most severe form of the disease, can lead to chronic headaches, epilepsy, intracranial hypertension and other neurological manifestations. For these reasons, the taeniasis/cysticercosis complex (TCC) caused by *T. solium* represents a substantial health and economic burden and is among the leading causes of acquired epilepsy in endemic regions of Latin America, sub–Saharan Africa, and Asia [1,6,7].

In light of this, the WHO set a goal to eliminate taeniasis/cysticercosis in its 2016–2022 action plan [4,8]. The current global strategy for 2021–2030 now focuses on the control of TCC through integrated interventions, including community health education, pig vaccination, preventive chemotherapy, and other coordinated measures [1]. However, a persistent challenge in endemic countries is the difficulty of identifying tapeworm carriers, despite their pivotal role in sustaining transmission; these individuals are frequently diagnosed only after developing symptomatic NCC [3,9].

Macroscopic and microscopic detection of *Taenia* spp. proglottids and eggs in faecal samples remains the most commonly employed diagnostic approach. However, this method exhibits limited diagnostic sensitivity (ranging from 38% to 69%), owing to intermittent proglottid shedding and the morphological similarity of eggs among *Taenia* species. Moreover, accurate identification requires specialised experienced personnel [10,11,12,13]. Coproantigen tests—which detect parasite metabolic products independently of egg or proglottid presence—offer an alternative, but they face challenges in differentiating between *T. solium* and *T. saginata*, potentially leading to undetected *T. solium* carriers and the persistence of local transmission [11,14]. Despite its relatively low sensitivity, microscopy remains the most widely available diagnostic tool for taeniasis [15].

Molecular diagnostic methods based on PCR amplification of *T. solium* DNA have also been developed demonstrating high sensitivity (99%) and specificity (94%) [12]. While these characteristics make PCR a reliable diagnostic tool, its elevated cost and requirement for well-equipped laboratories constrain its routine use in resource-limited, endemic settings [11,16].

Given the need for rapid, field-deployable diagnostic tests with robust performance, recombinant proteins and peptides have emerged as valuable components in the development of immunodiagnostic assays for taeniasis [17]. One promising approach involves the use of excretory/secretory (ES) proteins of the parasite as potential diagnostic targets [10,17]. However, knowledge regarding the immunogenic properties of these proteins remains limited. Therefore, the aim of this study was to identify *T. solium* proteins with high immunogenic potential, to express and purify them in a mammalian expression system, and ultimately to contribute to the development of improved diagnostic tools for the detection of carriers—a cornerstone for the effective control of this parasitic complex.

## 2. Results

### 2.1. Selection of T. solium Sequences

**Figure 1:**
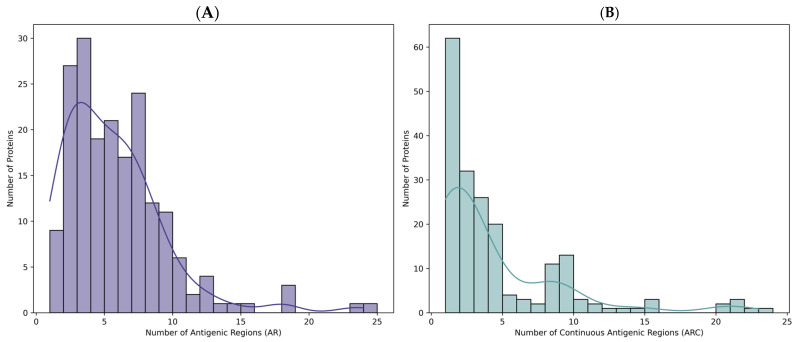
Distribution of antigenic regions. (**A**) continuous antigenic regions. (**B**) predicted with BepiPred-2.0 server.

To assess the immunogenic potential of the 586 *T. solium* protein sequences reported in the NCBI database, a B-cell epitope prediction analysis was performed. Following data collection and processing via the prediction server, the proteins were categorised according to the number of predicted antigenic regions (Figure 1A)—including those with no continuous antigenic regions (RAc = 0) within their sequences. Notably, 29.35% of the dataset did not exhibit any antigenic or continuous antigenic regions. Among these, 163 out of 172 proteins were identified as partial sequences, predominantly corresponding to mitochondrial proteins with incomplete information.

After the in silico evaluation of antigenic region abundance, the dataset was refined. A total of 396 proteins were excluded—172 lacking continuous antigenic regions (Figure 1B), 116 mitochondrial proteins, 79 partial sequences, 12 putative proteins, 14 hypothetical proteins, and 3 unnamed or uncharacterised entries.

A cut-off value of 81.3 was established for the IAARc (Index of Abundance of Continuous Antigenic Regions) to identify *Taenia solium* proteins with the highest immunogenic potential. Proteins exceeding this threshold demonstrated the greatest density of predicted epitopes. Given the potential application of these proteins in identifying taeniasis cases, further analysis focused on the subset of 190 proteins that exhibited the highest abundance of antigenic regions.

**Figure 2:**
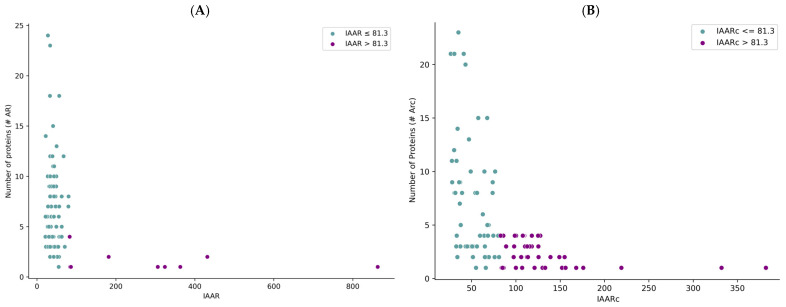
Classification of 190 *Taenia solium* proteins. (**A**) by Abundance Index of Antigenic Region. (**B**) by Abundance Index of Continuous Antigenic Region.

Among these, proteins were filtered according to their reported association with specific parasitic stages. Nine proteins were associated with the adult stage—102 corresponded to the cysticercus stage—and for 22 proteins the developmental stage could not be determined from the available metadata (Figure 2).

**Figure 3:**
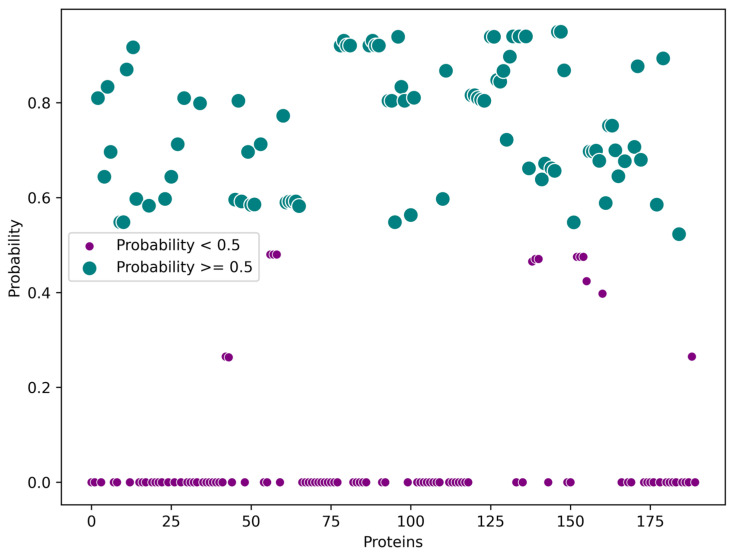
Proteins included and excluded based on the criteria established for the analysis performed through SignalP-5.0.

Due to the limited number of proteins linked to the adult stage, subsequent selection focused on the presence of signal peptides—as predicted using the SignalP 5.0 server (Figure 3).

A total of 88 protein sequences were identified with predicted signal peptides, suggesting their potential involvement in the excretory/secretory (ES) system of the parasite. Notably, the number of ES proteins identified in this study surpasses that reported in the previous literature.

Finally, using the metadata available for each protein in the NCBI database, redundant sequences and isoforms were removed. This curation step resulted in the selection of 22 *Taenia solium* proteins with in silico-characterised immunogenic potential and likely involvement in the excretory/secretory pathway.

### 2.2. Genetic Construction and Expression of T. solium Sequences

The designed genetic construct comprised: a cytomegalovirus (CMV) promoter, a start codon, a signal peptide sequence, five selected *T. solium* peptide sequences, a Strep-tag II sequence, and a picornavirus 2A peptide sequence—enabling translational self-cleavage and permitting polycistronic expression of different recombinant protein variants. Four distinct expression products were predicted, based on the number of incorporated *T. solium* sequences: the first with a molecular weight of 14.6–16.5 kDa; the second, 22.2–24.2 kDa; the third, 31.1–33.1 kDa; and the final version—39.2 kDa—encompassing all five peptides.

**Figure 4:**
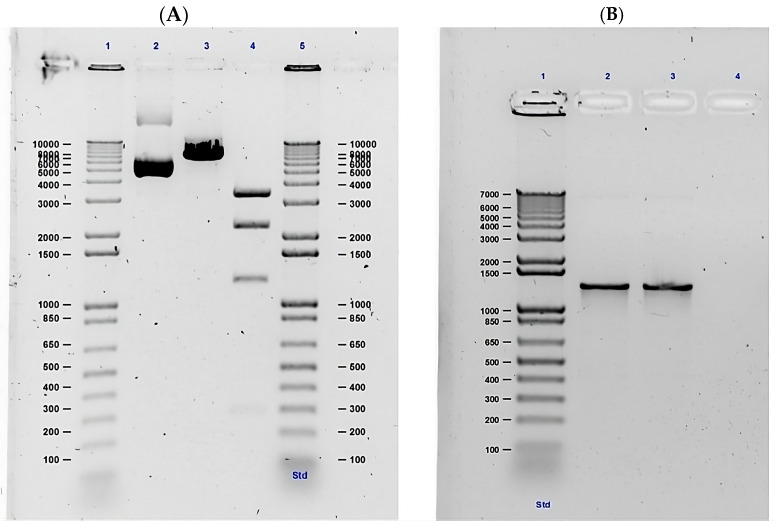
Verification of the *T. solium* polycistronic expression construct by restriction and sequencing: (**A**). Construct verification by restriction assay. Lane 1 and 5: Molecular weight marker of 1 Kb. Lane 2: undigested plasmid. Lane 3: digestion with PstI I (Thermo Scientific) that generates a cut in position 3742 with a weight of 7042 bp. Lane 4: digestion with Apal I (NEB) which makes three cuts in position 2029 (3327 bp), 5356 (1246 bp) and 6602 (2469 bp). (**B**). Construct verification by ONT sequencing. Lane 1: Molecular weight marker of 1 Kb. Lane 2 and 3: Amplification of the insert of the construct with the estimated weight (1293 bp). Lane 4: No template control.

Following successful transformation of the plasmid into *E. coli*, the construct was confirmed by restriction enzyme digestion—yielding fragment sizes consistent with the in silico-predicted molecular weights (Figure 4A). In addition, PCR amplification of the insert produced a band of the expected size (~1293 bp), as shown in Figure 4B. Amplicons were purified using a 0.7:1 bead-to-sample ratio, yielding 19.1 ng/µL of high-purity DNA for sequencing library preparation.

Nanopore sequencing revealed 100% identity with the reference sequence—with a sequencing depth exceeding 1000×—thereby confirming the integrity and accuracy of the construct.

**Figure 5:**
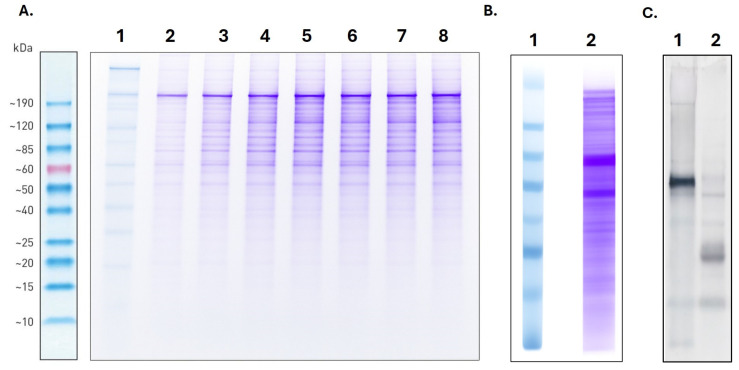
Recombinant proteins expression. (**A**). Expression of *T. solium* recombinant proteins. Lane 1: Molecular weight marker of 6–180 kDa (BenchMark™ Pre-stained Protein Ladder). Lane 2–8: Supernatant of the follow to the transfection assay with the recombinant proteins of *Taenia solium* on the day +1 of transfection to +7 day. (**B**,**C**). Western blot of the *T. solium* recombinant proteins. Lane 1: Molecular weight marker of 6–180 kDa. Lane 2: Supernatant of the day +6 of transfection on Coomassie stain (**B**) and recombinant proteins detected by WB (**C**).

Suspension cells were successfully cultured, with daily monitoring after thawing to ensure optimal conditions—maintaining cell viability above 90% and maintaining cell density below 3 × 10^6^ cells/mL. Once the sequence of the genetic construct was confirmed to be correctly synthesized within the plasmid, and sufficient plasmid DNA was obtained (200–500 ng/μL, A260/A280 > 1.8, and A260/A230 > 2.0), the transfection assay was carried out. The results indicated that day 6 post-transfection represented the optimal time point for recombinant protein production (Figure 5A). Specific versions of the recombinant proteins were successfully detected by Western blot through recognition of the Strep-tag II by the corresponding antibody (Lane 2, Figure 5C)—in contrast to the total proteins detected in the supernatant by Coomasie stain (Lane 2, Figure 5B).

### 2.3. Purification of T. solium Proteins

**Figure 6:**
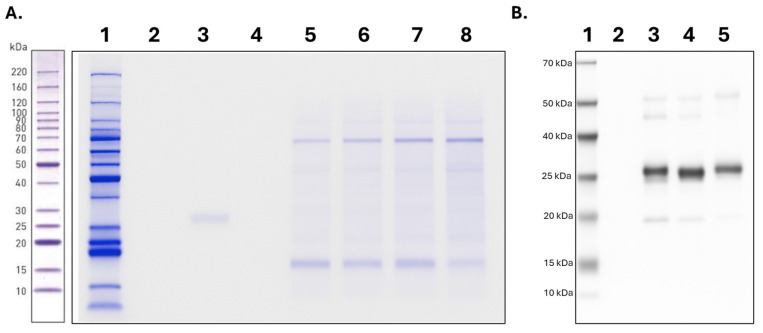
Purification of proteins. (**A**). Purification of the recombinant proteins. Lane 1: Molecular weight marker of 10–220 kDa (BenchMark™ Protein Ladder). Lanes 2 and 3: Elution one and two of the recombinant proteins Negative control of transfection. Lanes 4 and 5: Last and first wash. Lane 6: superflow of the pass of the resin. Lane 7: Supernatant with avidin. Lane 8: Supernatant of 6+ transfection day. (**B**). Western blot of the purified proteins. Lane 1: Molecular weight marker of 6–180 kDa. Lane 2: Negative control of transfection. Lane 3: Supernatant before the purification assay. Lanes 4 and 5: First and second elution of the recombinant proteins.

After confirming the expression of the recombinant proteins, purification with Strep-Tactin resin enabled the isolation of two recombinant protein variants—with estimated molecular weights of approximately 21 and 30 kDa—as observed in lanes 7 and 8 of the elution (Figure 6A). Additional predicted bands were detected in a subsequent SDS-PAGE experiment using the silver staining method; however, their abundance was insufficient for proper visualization. The purification process was monitored by evaluating the supernatant prior to purification (lane 3), the column flow-through (lane 4), and the initial and final wash steps (lanes 5 and 6, respectively).

Finally, Western blot analysis confirmed the presence of the two recombinant proteins through specific detection of the molecular tag, using a commercial anti-Strep-tag II antibody (Figure 6B).

## 3. Discussion

For the diagnosis of *Taenia solium* carriers, the most effective approach—as previously discussed—is the coproantigen test. This method enables early detection and the evaluation of treatment efficacy without requiring the presence of eggs or proglottids, as is the case with microscopy or copro-DNA tests. Its principal advantage lies in the continuous availability of antigens resulting from the parasite’s metabolism [11,14].

Most coproantigen studies have employed crude extracts or culture supernatants from the parasite to generate antibodies in animal models—subsequently used to directly detect the parasite in stool samples [14,18,19]. However, these approaches face several limitations, including issues in reproducibility, purification and standardisation, as well as the risk of cross-reactivity with antigens from other parasites. In contrast, serodiagnostic methods have advanced through the use of excretory/secretory (ES) antigens and recombinant proteins, which can overcome many of the aforementioned limitations [10,17].

Among the *Taenia* studies reported in the literature on recombinant protein expression, most focus on metabolic proteins [20,21], proteins involved in cell division [22], vaccine development [23], or the diagnosis of neurocysticercosis [2,24,25,26]. Other researchers have investigated recombinant proteins from related *Taenia* species—including *T. pisiformis*, *T. multiceps*, and *T. taeniaeformis*—for the diagnosis of parasitic diseases in animals, typically employing prokaryotic expression systems [27,28,29].

For other helminths—and in parasitology more broadly—the use of recombinant DNA technology has been extensively reported as a valuable strategy to improve diagnostic tools. Recombinant proteins provide a consistent and renewable source of antigen, facilitating test standardisation and enabling large-scale production [30]. This advantage opens the possibility of developing rapid, easy-to-use diagnostic kits suitable for large-scale surveys in endemic areas—once the recombinant antigen has been validated as highly sensitive and specific [31].

Currently, the selection of target proteins integrates parasitological, genomic, transcriptomic, and proteomic data—often supported by a range of bioinformatic approaches, as in this study. In the case of helminths, *E. coli* has been the most frequently used expression system for producing recombinant proteins to evaluate immunomodulatory properties in vitro and in experimental models. This preference stems from the system’s low cost, ease of manipulation, and high yield. However, it has important limitations—notably the absence of post-translational modifications and the potential for incorrect protein folding—factors that may compromise biological function [32,33].

The immunological potential of the 22 *T. solium* proteins evaluated in this study, based on the IAARc (Index of Antigenic Abundance in Regions) values, was comparable to that reported for excretory/secretory (ES) proteins of *T. solium*, the secretome of other helminths, and proteins already applied in diagnosis [34]. Our approach stands out by prioritising sequences with predicted exposed B-cell epitopes and high IAARc values. Furthermore, the selected proteins are likely to be part of the parasite’s excretory/secretory system—molecules continuously released during its metabolism and therefore accessible to the host immune system [34,35]. This increases their diagnostic potential, since detection does not depend on the presence of intact eggs or proglottids, unlike conventional parasitological or copro-DNA methods [11].

A complementary analysis conducted in December 2023 revealed an increase in the number of available *T. solium* protein sequences, reaching a total of 758. After excluding sequences classified as hypothetical, partial, unnamed, uncharacterized or mitochondrial, 240 proteins remained. Further filtering led to the selection of 190 proteins, resulting in a discrepancy of 50 sequences—excluded due to the absence of predicted antigenic regions. These sequences corresponded primarily to calreticulin chains, patented proteins, and recombinant proteins. Their exclusion is therefore unlikely to significantly affect the overall results or the principal conclusions of this study.

The recombinant proteins in this study were successfully expressed in a soluble fraction, which facilitated recovery and improved reproducibility and efficiency in downstream processing. Although the chosen mammalian expression system entails higher costs than bacterial systems, it offers several advantages. The use of a chemically defined medium reduces background protein contamination—as observed in Figure 5A and Figure 6A—and ensures that the recombinant proteins are produced with appropriate tertiary structure and relevant post-translational modifications, such as glycosylation—an essential feature often absent or misrepresented in *E. coli* systems [36].

Moreover, this expression platform proved cost-effective in the context of multiprotein production. By enabling the simultaneous expression of several proteins in a single construct, it obviates the need for separate transfections and complex protein refolding procedures required after purification in bacterial systems—as previously reported for *Fasciola hepatica* [37]. In addition, *E. coli* expression systems have shown low success rates (6–21%) in the large-scale production of *Plasmodium falciparum* recombinant proteins [38], further supporting the suitability of mammalian cells for expressing helminth antigens with diagnostic or immunological potential.

Our results are comparable to those reported by Elton and collaborators [39]. As with *T. solium*, the complete genome of *Babesia microti* was sequenced, enabling the identification and expression of extracellular proteins of interest owing to their direct exposure to the host’s humoral immune system—akin to the excretory/secretory (ES) proteins in our study. The 3609 coding regions of *B. microti* were analysed in silico, with a focus on identifying proteins containing transmembrane domains, signal peptides, and glycosylphosphatidylinositol (GPI) anchors. These were predicted using Phobius, PredGPI, and SignalP v4.1. Proteins lacking signal peptides, showing homology with intracellular organelles, or displaying high similarity to other *Babesia* or *Plasmodium* species were excluded. In addition, proteins not expressed in transcriptome data from mouse and hamster infection models were removed—retaining only those expressed during the blood stage. The authors also undertook a manual annotation of *gene loci* by aligning transcriptomic data to the *B. microti* genome. This comprehensive analysis underscores the importance of rigorous in silico protein selection to maximise the immunological potential of candidates for downstream applications.

In their work, a panel of 54 proteins was expressed individually using constructs that included codon-optimized sequences for expression in human cells, the rat CD4 domains 3 and 4 as an antigenic tag, an enzymatically biotinylatable sequence, and a 6xHis tag—following a strategy previously applied to *Plasmodium* protein libraries. The plasmids were expressed in HEK 293 suspension cells, and the biotinylated proteins were collected six days post-transfection, detected via ELISA, and purified using the His tag for subsequent immunological and functional characterization [39].

In contrast, our study achieved the expression of four recombinant proteins within a single construct—thereby optimising both time and resources. Additionally, we employed the Strep-tag II system rather than the His-tag system. The Strep-tag has been recommended in the literature for its superior compatibility with mammalian cell culture media and reagents, its specific elution conditions, and its ability protect against protein degradation. It also yields a higher purity level (~95%) compared with the ~80% typically achieved with the His tag. This advantage has been corroborated by Lichty and collaborators [40], who observed greater purity using the Strep-tag system in extracts from *E. coli*, yeast, *Drosophila*, and HeLa cells—with good yields and moderate cost [41].

Similarly to our study, previous works have proposed using the density of epitopes within a single protein molecule as a metric for evaluating its antigenicity and immunogenicity [35]. In particular, Gomez et al. [34] introduced the concept of Abundance of Antigenic Regions (AAR), which quantifies the number of predicted antigenic regions relative to the sequence length. When applying this metric to evaluate the antigenic potential of the *T. solium* secretome, they found that the AAR values for secreted proteins resembled those of experimentally validated antigenic proteins—and differed significantly from those of non-excretory/secretory (ES) proteins encoded in the *T. solium* genome.

This type of comprehensive in silico analysis provides valuable insights for identifying novel proteins of therapeutic, diagnostic, and immunological relevance. It exemplifies how epitope density, expressed as AAR, can serve as a practical and informative metric for estimating immunogenic potential at the genomic level (see Supplementary Materials S1 for accession number, description and name of the top selected). Notably, while Gomez et al. [34] employed the BepiPred-1.0 prediction tool, the present study utilised the updated BepiPred-2.0 version. This newer tool delivers improved performance by integrating predictions of surface accessibility and secondary structure—offering greater accuracy when combined with additional bioinformatic methods such as SignalP.

A limitation of the present study, however, is the reliance on in silico predictions for selecting candidate proteins. Although tools such as BepiPred-2.0 and SignalP-5.0 provide useful information, they remain predictive models and cannot fully account for factors such as protein stability in biological samples, post-translational modifications or variability in immune responses. Consequently, the immunogenic potential suggested by these analyses must be corroborated through experimental and clinical validation.

Given the exploratory nature of the present study, a key limitation is the absence of cross-reactivity testing with phylogenetically related parasites—such as *Taenia saginata*—as well as smaller cestodes, including *Hymenolepis* spp. and Dipylidium caninum. Future investigations should address this by performing cross-reactivity assays, followed by ELISA validation using well-characterized positive coproantigen samples from these parasites, which are intestinal and occur with appreciable frequency in our setting. The inclusion of appropriate negative controls—together with representative clinical panels and the calculation of sensitivity and specificity—will be essential to ensure the robustness and external validity of the assay.

It is also important to recognise the broader limitations imposed by the scarcity of commercial and widely adopted diagnostic tools for tapeworm detection. This situation largely reflects their classification as neglected diseases, typically restricted to vulnerable populations in developing regions. Moreover, the inherent biology of the parasite—particularly the intermittent excretion of Taenia eggs in stool—further constrains diagnostic sensitivity. These challenges underscore the urgent need for complementary diagnostic strategies, including antigen detection and molecular assays, which should be prioritised in future research to enhance diagnostic accuracy and broaden the clinical applicability of novel approaches.

## 4. Materials and Methods

### 4.1. Taenia solium Protein Sequences

Protein sequences of *Taenia solium* were retrieved from the National Center for Biotechnology Information (NCBI) protein database [42] using the query “*Taenia solium*”. As of 6 February 2020, a total of 586 protein sequences were available and downloaded in FASTA format for downstream analysis. Associated metadata for each protein was exported as an XML file.

B-cell epitope prediction was performed using the BepiPred-2.0 server [43], based on primary amino acid sequences. Structural conformation and epitope surface accessibility were assessed through the advanced output feature of BepiPred-2.0 [44]. Antigenic regions were defined as contiguous stretches of at least six amino acids predicted to constitute B-cell epitopes.

Relevant data—including the number of predicted antigenic regions, structural features and epitope exposure—were compiled into a custom database. The Abundance of Antigenic Regions (AAR) was calculated following the method described by Gómez et al. [34], using the following equation:

$$Xp=\frac{Lp}{Ap}$$

where:

*Xp* denotes the relative Abundance of Antigenic Regions in protein *p*,
*Lp* the sequence length in protein *p*,
*Ap* the number of antigenic regions in protein *p*.

Additionally, we defined a novel parameter: the Abundance of Continuous Antigenic Regions (AARc), which accounts for the predicted structural conformation of the protein and the surface exposure of epitopes. This parameter includes only those antigenic regions not disrupted by the protein’s three-dimensional structure, following the approach described by Wang et al. [35].

Following epitope prediction, all proteins lacking any predicted antigenic regions were excluded. We then conducted a descriptive statistical analysis of two indices: the IAAR (Index of Abundance of Antigenic Regions) and the IAARc (Index of Abundance of Continuous Antigenic Regions). For each index, we determined the mean, interquartile range (IQR), and overall variation—to characterise the distribution of total and structurally continuous antigenic regions across the *T. solium* proteome.

To ensure biological relevance, mitochondrial proteins—together with those annotated as partial, hypothetical, or putative—were excluded due to the lack of experimental validation or incomplete functional annotation.

After this curation step, a total of 190 protein sequences were retained. A subsequent literature review was undertaken to identify the developmental stage (s) in which these proteins are expressed. From this refined dataset, proteins with metadata indicating expression in either the egg or adult stages—according to GenBank annotations—were selected for further analysis.

### 4.2. Signal Peptide Prediction

The presence of signal peptides and the location of their cleavage sites in *Taenia solium* proteins were predicted using the SignalP 5.0 server [45]. This analysis aimed to identify proteins potentially involved in the parasite’s excretory/secretory pathways. The predicted secretory pathway type and associated probability scores were compiled into a structured database.

Information on the predicted presence of Signal Peptidase I or II, as well as the likelihood of a cleavage site, was collected and organised in a Microsoft Excel spreadsheet. Proteins were initially filtered using the server’s default threshold (<0.5). Only those sequences with probability scores greater than 0.5—indicating a high likelihood of secretion or cleavage—were retained for further analysis.

**Figure 7:**
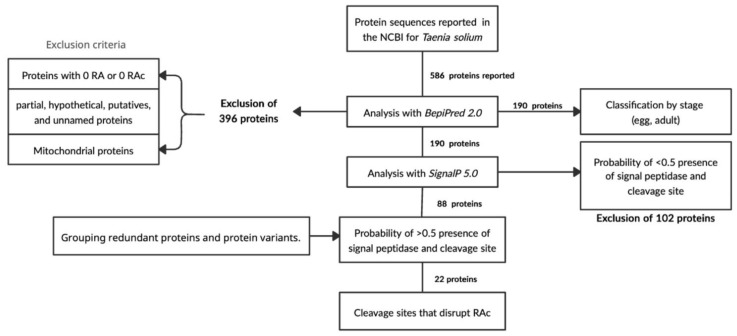
Bioinformatic workflow of the analysis and selection of the *Taenia solium* sequences reported on the NCBI.

A visual representation of this bioinformatics workflow is presented in Figure 7.

### 4.3. Genetic Construction and Verification

To enable polycistronic expression of selected *Taenia solium* peptide sequences in a eukaryotic system, a genetic construct was designed using the pcDNA3.4-TOPO vector as a backbone. The peptide-coding sequences were selected on the basis of published literature and were synthesised de novo using the GeneArt service (Thermo Scientific).

Codon optimization for *Homo sapiens* and *Cricetulus griseus* was performed to enhance gene expression in HEK and CHO cell models (codon adaptation index [CAI] > 0.9). Negative cis-acting elements (e.g., splice sites and TATA boxes) that could interfere with expression were removed. The GC content was adjusted to improve mRNA stability. The resulting 1041 bp DNA fragment was inserted into the pcDNA3.4-TOPO vector downstream of the CMV promoter. The construct also included a Strep-tag—facilitating recombinant protein purification and identification by Strep-tag Western blotting.

The synthesised expression vector was verified by Sanger sequencing and subsequently cloned into *E. coli* using the One Shot TOP10 Chemically Competent Cells Kit (Thermo Scientific, Rockford, IL, USA), in accordance with the manufacturer’s instructions.

Transformed colonies were selected by antibiotic resistance on agar plates. A single colony was isolated and cultured in Luria–Bertani broth supplemented with ampicillin (100 μg/mL) for 36 h at 37 °C and 100 rpm. Plasmid DNA was extracted using the GeneJET Plasmid Midiprep Kit (Thermo Scientific), following the manufacturer’s protocol. DNA purity was assessed via spectrophotometry using a NanoDrop 2000 (Thermo Scientific), and the eluate was stored at −40 °C.

Construct verification after bacterial transformation and propagation was performed by double digestion with restriction enzymes—as recommended by the manufacturer—and visualised by electrophoresis on 1% agarose gel in Tris–borate–EDTA (TBE) buffer. In parallel, the presence and identity of the insert were confirmed by PCR amplification, followed by sequencing using Oxford Nanopore Technology (Oxford Nanopore Technologies, Oxford, UK).

PCR was carried out using universal primers targeting flanking regions within the vector (pcDNA3.4):

Forward primer: 5′-CGCAAATGGGCGAGGCGTG-3′

Reverse primer: 5′-CAACATAGTTAAGAATACCAGTC-3′

Each 20 μL PCR reaction contained: 2× reaction buffer, 2 mM MgCl_2_, 0.4 U/μL recombinant Taq DNA polymerase, 20 μM dNTPs, 1 μM of each primer (synthesised by Macrogen, Seoul, Korea), and 3 ng/μL plasmid DNA as template. Amplification was conducted on a C1000 thermal cycler (Bio-Rad, Hercules, CA, USA) with the following conditions: 98 °C for 2 min; 40 cycles of 98 °C for 10 s, 63 °C for 15 s, and 72 °C for 1 min. PCR products were analysed by electrophoresis on 1% agarose gel in TBE buffer at 80 V for 1 h and visualised using a GelDoc™ XR + imaging system with Image Lab software version 3.0.1.14 (Bio-Rad Hercules, CA, USA).

Amplicon purification, quantification, library preparation, and sequencing were performed according to the protocol described by Tyson [46], using the SQK-LSK109 kit (Oxford Nanopore Technologies) and R9.4.1 flow cell chemistry. Sequencing was carried out on a MinION Mk1C device (Oxford Nanopore Technologies, Oxford, UK) for 24 h. Raw FAST5 files were processed with Guppy (ONT) for basecalling to generate FASTQ files. Finally, sequence reads were mapped to the reference sequence of the original construct for confirmation.

### 4.4. Expression of Taenia solium Recombinant Proteins

Gibco™ Expi293F suspension cells were cultured in Expi293™ Expression Medium without the addition of antibiotics or antifungals, following the manufacturer’s instructions and standard laboratory protocols. A mammal cell line was chosen in view of the predicted post-translational modification sites in the recombinant protein—including glycosylation, which cannot be reproduced in bacterial expression systems. The culture was passaged at least three times to ensure stability and to achieve a cell viability of ≥90%, as determined by trypan blue exclusion assay.

Cells were maintained under the following conditions: 37 ± 0.5 °C, 8% CO_2_, 120 ± 5 rpm, in a humidified atmosphere. Transient transfection was performed using the ExpiFectamine™ 293 Transfection Kit (Thermo Scientific), in accordance with the manufacturer’s protocol. The DNA–Opti-MEM™ mixture was filtered through a 0.22 µm sterile membrane to ensure aseptic conditions prior to transfection.

Soluble recombinant proteins were harvested by centrifugation of the culture supernatant. The clarified supernatant was supplemented with 10% glycerol and cOmplete™ Protease Inhibitor Cocktail (Roche, Basel, Switzerland) at 1× concentration to preserve protein integrity. Samples were aliquoted and stored at −80 °C until further use.

### 4.5. Electrophoresis and Western Blot

Denaturing SDS–PAGE was performed using a 12.5% polyacrylamide gel. Following electrophoresis, the gel was stained with Coomassie Brilliant Blue and subsequently destained to verify the molecular weights of the expressed proteins. In parallel, proteins from a duplicate gel were transferred overnight at 20 V to an Immobilon-P membrane (Millipore, Burlington, MA, USA).

The membrane was briefly stained with Ponceau S solution to confirm successful transfer, then washed three times with PBS 1× containing 0.1% Tween-20 (PBS-T) at 30 rpm. Non-specific binding sites were blocked by incubating the membrane in PBS 1× supplemented with 5% skimmed milk for 1 h at 20 rpm.

The primary antibody, Anti-Strep-tag II (Abcam, Cambridge, UK), was diluted 1:5000 in the blocking solution and incubated with the membrane overnight at 2–8 °C under gentle agitation (20 rpm). After washing three times with PBS-T, the membrane was incubated for 2 h at 18–20 °C with a secondary antibody: Anti-Rabbit IgG (H + L), conjugated to alkaline phosphatase (Bio-Rad, Hercules, CA, USA), diluted 1:250 in blocking solution.

Detection was performed using a chromogenic substrate containing nitro-blue tetrazolium (NBT) and 5-bromo-4-chloro-3-indolyl phosphate (BCIP), enabling the visualisation of immunoreactive bands.

### 4.6. Purification of Taenia solium Recombinant Proteins

**Figure 8:**
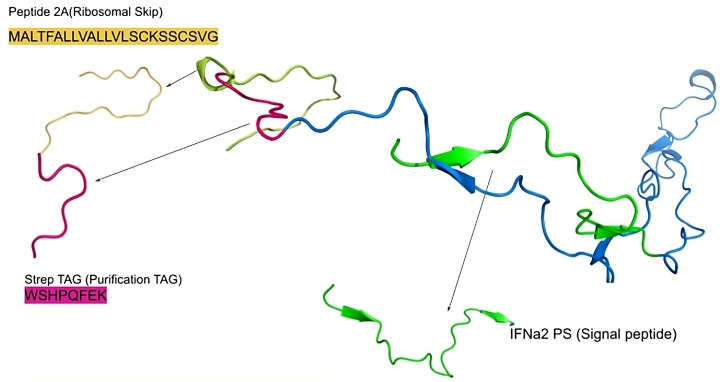
Prediction of the tertiary structure of the expressed recombinant protein.

The recombinant proteins (Figure 8), each containing a C-terminal Strep-tag II, were purified by affinity chromatography using Strep-Tactin^®^ 4FF resin (MyBioSource San Diego, CA, USA). To prevent inactivation of the resin, biotin and biotinylated proteins present in the culture supernatant were masked using egg white avidin, as recommended by the manufacturer (IBA Lifesciences, Göttingen, Germany).

All purification steps were conducted at 2–8 °C to minimise protein degradation and prevent bubble formation. The clarified supernatant was filtered through a 0.45 µm PVDF membrane prior to application onto the resin, thereby ensuring sterility and removing particulates.

The resin was equilibrated with binding buffer before applying the protein-containing supernatant. The column was washed with binding buffer until the absorbance at 280 nm—measured using a NanoDrop 2000 (Thermo Scientific)—approached baseline levels. Elution of recombinant proteins was carried out using a desthiobiotin-containing buffer (Santa Cruz Biotechnology, Dallas, Texas, USA), in accordance with the resin manufacturer’s guidelines.

## 5. Conclusions

In this study, we present a comprehensive framework for the selection, expression, and purification of recombinant proteins with in silico-predicted immunological potential, preserved within a eukaryotic expression system. The ultimate aim is to employ these proteins in the near future for the development of direct immunological assays designed for the specific detection and monitoring of *Taenia solium* infections using clinical samples to confirm the utility of the recombinant proteins. Such diagnostic tools could play a pivotal role in interrupting the transmission cycle of the disease over time.

Furthermore, this work contributes to one of the key strategies proposed by the World Health Organization: the development of sensitive and specific diagnostic tools for taeniasis, as part of the global initiative to control—and ultimately eliminate—the *Taeniasis/Cysticercosis* complex caused by *T. solium*.

## Data Availability

The original contributions presented in this study are included in the article/Supplementary Materials. Further inquiries can be directed to the corresponding author.

## Supplementary Materials

The following supporting information can be downloaded at: https://www.mdpi.com/article/10.3390/ijms26199585/s1.
