# Supplementary material for: From Bioinformatics Analysis to Recombinant Expression: Advancing Public Health with Taenia solium Proteins
Source: Int J Mol Sci. 2025 Oct 1;26(19):9585. doi: 10.3390/ijms26199585 (PMC12524493; doi:10.3390/ijms26199585)
Supplement: Supplementary file 1 [file ijms-26-09585-s001.zip › ijms-3838070-supplementary.pdf]

Supplementary Materials (SM)

SM S1. Accession number, description and name of the top selected proteins of *Taenia solium* with predicted antigenic potential based on *in silico* analysis

Accession	Description	Protein name
AEP03196.1	CyDA variant 1 [*Taenia solium*]	**150 kDa**
AEP03200.1	RS2 [*Taenia solium*]	
ABI23958.1	variant [*Taenia solium*]	**B1**
AEP03195.1	variant 2 [*Taenia solium*]	
AAK52725.1	**calcium-binding protein calreticulin precursor [*Taenia solium*]**	
BAH03395.1	like cysteine peptidase [*Taenia solium*]	**Cathepsin L**
AAS00027.1	like cysteine proteinase [*Taenia solium*]	
AIM55117.1	**cystatin *[Taenia solium]***	
ABI20728.1	cysticercosis antigen [*Taenia solium*]	**10 kDa**
AAF06716.1	cysticercosis specific antigen [*Taenia solium*]	
ABI20733.1	b1 [*Taenia solium*]	**E/S Antigen**
ABI20730.1	m13 [*Taenia solium*]	
ABI20729.1	low molecular weight antigen 1[*Taenia solium*]	
BAB18643.1	low molecular weight antigen 1 variant 1 [*Taenia solium*]	
BAB18644.1	low molecular weight antigen 2 [*Taenia solium*]	
BAB18645.1	low molecular weight antigen 2 variant 1 [*Taenia solium*]	
BAB18646.1	low molecular weight excretion-secretion antigens b1 [*Taenia solium*]	
CAD44556.1	low molecular weight excretion-secretion antigens m4 [*Taenia solium*]	
CAD44557.1	m13h low molecular weight excretion-secretion antigen [*Taenia solium*]	
CAD44555.1	m13h variant [*Taenia solium*]	
ABI23957.1	m13h variant 1 [*Taenia solium*]	
AEP03197.1		
AHH45014.1	1 [*Taenia solium*]	**Fasciclin**
AHH45015.1	2 [*Taenia solium*]	
CAO82075.1	**HP6 protein *[Taenia solium]***	
AEO45118.1	protein 1 [*Taenia solium*]	**Hydrophobic ligand-binding protein**
AEO45119.1	protein 2 [*Taenia solium*]	
AAW88556.1	a [*Taenia solium*]	**TSO31**
ABH07373.1	a [*Taenia solium*]	
AAW88557.1	b [*Taenia solium*]	
ABH07374.1	b [*Taenia solium*]	
AAW88558.1	c [*Taenia solium*]	
ABH07375.1	c [*Taenia solium*]	
AAW88559.1	d [*Taenia solium*]	
ABH07376.1	d [*Taenia solium*]	
AAN74962.1		**TSOL16**

AAN74964.1	A [*Taenia solium]*	
AAN74963.1	A [*Taenia solium*]	
	B [*Taenia solium*]	
AAM82156.1	**oncosphere-specific antigen [*Taenia solium*]**	
CAD48845.1	B1 [*Taenia solium*]	**TS8 secreted antigen**
CAD48846.1	B2 [*Taenia solium*]	
CAD48847.1	B3 [*Taenia solium*]	
ATG83399.1	**serpin [*Taenia solium*]**	
ADP89566.1	**trypsin-like protein [*Taenia solium*]**	
AIM56899.1	TS14 [*Taenia solium*]	**TS4 Glycoprotein 14 kDa**
AAF67357.1	TS14 14 kDa glycoprotein [*Taenia solium*]	
ABI20731.1	TS14 14 kDa glycoprotein precursor [*Taenia solium*]	
AAD51764.2	TS14 14 kDa glycoprotein precursor [*Taenia solium*]	
AAF25005.1	TS14 14kDa diagnostic antigen [*Taenia solium*]	
AEP03201.1	TS14 variant 1a [*Taenia solium*]	
AEP03202.1	TS14 variant 2b [*Taenia solium*]	
AAD09326.1	TS18 [*Taenia solium*] activated oncosphere TSOL18	**TS18/TSOL18 Glycoprotein**
AAQ16679.1	TS18 18 kDa glucoprotein [*Taenia solium*]	
AQR06134.2	TS18 18 kDa vaccine antigen [*Taenia solium*]	
ABI20732.1	TS18 kDa glycoprotein TS18 precursor [*Taenia solium*]	
AAK21959.1	variant 1 [*Taenia solium*]	
AAK21960.1	variant 2 [*Taenia solium*]	
ADO86981.1	TS18 kDa oncosphere antigen [*Taenia solium*]	
AEP03203.1	variant 1 [*Taenia solium*]	
AEP03204.1	variant 2 [*Taenia solium*]	
AEP03205.1	variant 3 [*Taenia solium*]	
AEP05206.1	variant 4 [*Taenia solium*]	
AEP03207.1	variant 5 [*Taenia solium*]	
AAO65442.1	TSES33 [*Taenia solium*]	**TSES33**
CAX86983.1	TSES33 diagnostic antigen [*Taenia solium*]	
CAX86982.1	TSES38 diagnostic antigen [*Taenia solium*]	**TSES38**
AAM96903.1	TSES38 diagnostic protein [*Taenia solium*]	
AAK31938.1	1A [*Taenia solium*]	**TSO45/TSOL45**
AAK31940.1	1A [*Taenia solium*] activated oncosphere	
AAK31946.1	1B [*Taenia solium*] activated oncosphere	
AAK31939.1	4B [*Taenia solium*]	
AAK31944.1	4B [*Taenia solium*] activated oncosphere	
AAK31945.1	5B [*Taenia solium*] activated oncosphere	
AAM88223.1	A2 [*Taenia solium*]	
AAM88225.1	A5 [*Taenia solium*]	
AAM88220.1	B1 [*Taenia solium*]	
AAM88229.1	B10 [*Taenia solium*]	
AAM88222.1	B11 [*Taenia solium*]	

AAM88226.1	B2 [*Taenia solium*]	
AAM88221.1	B3 [*Taenia solium*]	
AAM88227.1	B8 [*Taenia solium*]	
AAM88228.1	B9 [*Taenia solium*]	
AAQ75030.1	W-4 [*Taenia solium*] glucoprotein	
AAQ75028.1	W-4B [*Taenia solium*] glucoprotein	
AAQ75029.1	W-4B [*Taenia solium*] glucoprotein	
AAQ83444.1	W-4B [*Taenia solium*] glucoprotein	
AAM88385.1	W-A12 [*Taenia solium*] activated oncosphere	
AHC68881.1	Wnt4 [*Taenia solium*]	
